# Prediction of advanced colonic neoplasm in symptomatic patients: a scoring system to prioritize colonoscopy (COLONOFIT study)

**DOI:** 10.1186/s12885-019-5926-4

**Published:** 2019-07-25

**Authors:** Fernando Fernández-Bañares, Ramon Clèries, Jaume Boadas, Josepa Ribes, Joan Carles Oliva, Antoni Alsius, Xavier Sanz, Eva Martínez-Bauer, Sara Galter, Mar Pujals, Marta Pujol, Patricia del Pozo, Rafel Campo

**Affiliations:** 1 Department of Gastroenterology, Hospital Universitari Mutua Terrassa, Plaza Dr Robert 5, 08221 Terrassa, Barcelona Spain; 2 Centro de Investigación Biomédica en Red de enfermedades hepáticas y digestivas (CIBERehd), Instituto Salud Carlos III, Madrid, Spain; 3 Pla Director d’Oncologia, IDIBELL, L’Hospitalet de Llobregat, Barcelona, Spain; 4 Department of Gastroenterology, Consorci Sanitari de Terrassa, Terrassa, Barcelona Spain; 5 CATLAB, Viladecavalls, Barcelona, Spain; 6 Department of Gastroenterology, Hospital Parc Taulí, Sabadell, Barcelona Spain

**Keywords:** Colorectal cancer, Advanced adenoma, Faecal immunological occult haemoglobin test, Fast-track colonoscopy

## Abstract

**Background:**

Fast-track colonoscopy to detect patients with colorectal cancer based on high-risk symptoms is associated with low sensitivity and specificity. The aim was to derive a predictive score of advanced colonic neoplasia in symptomatic patients in fast-track programs.

**Methods:**

All patients referred for fast-track colonoscopy were evaluated. Faecal immunological haemoglobin test (3 samples; positive> 4 μg Hb/g), and a survey to register clinical variables of interest were performed. Colorectal cancer and advanced adenoma were considered as advanced colonic neoplasia. A sample size of 600 and 500 individuals were calculated for each phase 1 and phase 2 of the study, respectively (Phase 1, derivation and Phase 2, validation cohort). A Bayesian logistic regression analysis was used to derive a predictive score.

**Results:**

1495 patients were included. Age (OR, 21), maximum faecal-Hb value (OR, 2.3), and number of positive samples (OR, 28) presented the highest ORs predictive of advanced colonic neoplasia. The additional significant predictive variables adjusted for age and faecal-Hb variables in Phase 1 were previous colonoscopy (last 5 years) and smoking (no, ex/active). With these variables a predictive score of advanced colonic neoplasia was derived. Applied to Phase 2, patients with a Score > 20 had an advanced colonic neoplasia probability of 66% (colorectal cancer, 32%), while those with a Score ≤ 10, a probability of 10% (colorectal cancer, 1%). Prioritizing patients with Score > 10, 49.4% of patients would be referred for fast-track colonoscopy, diagnosing 98.3% of colorectal cancers and 77% of advanced adenomas.

**Conclusions:**

A scoring system was derived and validated to prioritize fast-track colonoscopies according to risk, which was efficient, simple, and robust.

**Electronic supplementary material:**

The online version of this article (10.1186/s12885-019-5926-4) contains supplementary material, which is available to authorized users.

## Background

Colorectal cancer (CRC) is the third most common malignancy in men and the second most common in women, and the fourth leading cause of cancer-related deaths worldwide [1]. Despite progress made in recent years in the diagnosis and treatment of this disease, a significant improvement in survival at 5 years has not been shown, which persists around 50%. This is because over 80% of new cases are symptomatic patients, and the disease is advanced at the time of diagnosis. A significant percentage of CRC patients are diagnosed based on the presence of clinical symptoms associated with this malignancy [2–5]. It is therefore important to identify patients who have symptoms and/or signs of suspicion, so an early colonoscopy can be indicated. Recently, the proposed rule of 2-week wait (2WW) referral system for the NHS (UK) has been re-evaluated [6]. This system was proposed in 2000 to ensure that specialists in reference hospitals assess all patients with suspected CRC within 14 days after urgent referral by a primary care physician. This approach is based on the guidelines of the National Institute for Health and Care Excellence (NICE) criteria for suspected cancer [7]. However, different studies have shown the low sensitivity and specificity of the NICE criteria to refer for a 2WW program [8–11]. Although 77% of patients with CRC are referred by primary care physicians via urgent pathways, this system did not improve 5-year colorectal cancer survival [12]. This finding may be due to the fact that the symptoms when present very often indicate an advanced disease, and once a cancer became symptomatic, early treatment did not improve survival. Besides, the 2WW rule has been criticized, because of the low overall cancer detection rate, due to the poor specificity of the patient clinical symptoms (common with benign bowel diseases), resulting in overwhelming referral rates. The new NICE guidelines (July 2017) suggest using faecal occult blood test for clinical symptoms associated with a low probability of having CRC (PPV < 3%).

In this scenario, a number of CRC prediction models have been designed and validated in different settings [13]. These prediction models are calculated from mathematical equations based mainly on symptoms [14–16], and recently also on 1-sample faecal immunological test (FIT) [11, 17]. The patients included in those studies were symptomatic, but they did not always fulfil the criteria for a fast-track program. The diagnostic accuracy of these risk-scoring models is generally considered satisfactory and better than the existing referral criteria, but at present, they have not been widely recommended, perhaps as a consequence of the complicated mathematical equations required for their calculation.

In spite that a meta-analysis showed that in average-risk asymptomatic patients increasing the number of FIT samples did not affect the pooled performance characteristics of FITs for CRC [18], recent studies suggested that in symptomatic patients using either 2 or 3 tests provided the best discrimination for CRC [19–23].

Thus, the aim of the study was to derive and validate a predictive risk score to determine the pre-test-probability of advanced colonic neoplasia (ACN) in symptomatic patients with indication of a fast-track colonoscopy. In addition, to assess the accuracy for ACN diagnosis of a 3-sample FIT as compared to 1-sample FIT.

## Methods

The study protocol is available as a supplementary file (Additional file 1).

### Study population

All patients in the fast-track colonoscopy programs for CRC in the three participating hospitals were included in the period March-2014 to September-2016. Healthcare areas of these hospitals comprise a population of about 917,905 inhabitants. Patients with high-risk symptoms for CRC were sent for a full colonoscopy following the government’s program to expedite the diagnosis of CRC. All the colonoscopies in the health areas involved are performed at the endoscopic units of the three centres. Each unit performed more than 3,000 colonoscopies a year. Fast-track colonoscopy was requested mainly from primary care physicians or gastroenterology specialists in primary care medicine, but also from the hospital outpatient visits (different medical and surgical specialties). Inclusion criteria were as follows: 1. Age of 18 years or more; 2. Fulfilment of the criteria for a fast-track colonoscopy based on NICE guidelines [7]; 3. Signing informed consent. The exclusion criteria included pregnancy, asymptomatic individuals who were undergoing colonoscopy for CRC screening, patients with a previous history of colonic disease scheduled for a surveillance colonoscopy, patients requiring hospital admission, and patients whose symptoms had ceased within the three months before evaluation.

The clinical research ethics committees of the three centres approved the study and patients provided written informed consent.

### Interventions

Trained case managers (specialized nurses) conducted the patients’ personal interviews to assure a standardized and proper collection of faecal samples, the fulfilment of the inclusion and exclusion criteria, and the signing of informed consent. In all patients the following interventions were performed: 1. FIT on three different days in the week prior to colonoscopy; and 2. A patient consultation questionnaire including a detailed assessment of colorectal symptoms, personal and family history of polyps/CRC, smoking and consumption of medication increasing the risk of a gastrointestinal haemorrhage (NSAIDs, aspirin, anticoagulants). In the case of rectal bleeding, patients were instructed, if possible, to collect faecal samples on days that they did not have rectal bleeding. Faecal haemoglobin was analysed using iFOB kit (Linear Chemicals SL, Barcelona, Spain) (see detailed description of the test in Additional file 2) which is able to detect values of 4 μg Hb/g faeces. Quantitative values were recorded for each of the three samples from 4 to higher than 160 μg Hb/g faeces.

A full colonoscopy with iv sedation with colon biopsies and/or polypectomy, if necessary, was performed on all evaluated patients. Endoscopists involved in the study had more than 2 years of experience from routine clinical colonoscopies, undertaking at least 200 procedures per year, with caecal intubation rates over 97%. The cleaning degree (Boston scale), results of the exploration, and pathology studies were registered. Colonoscopies with insufficient preparation (Boston scale = 1 or less in one colonic segment) were repeated. In the case of multiple polyps, the number of polyps, the size of the largest, and the most advanced pathology were registered. If CRC was recognized, its stage (TNM classification) and location (right, transverse, or left colon) was recorded.

### Main outcome

The main outcome was ACN detection, which was defined as the presence of CRC or advanced adenoma (AA) (> 1 cm or high-grade dysplasia or villous component). Intramucosal carcinoma (Tis) was considered as AA.

### Derivation and validation cohort

The rule of thumb of 10 events per variable was used to obtain a derivation cohort, assuming that the logistic regression model may account for between 10 and 15 dummy predictor variables [24]. In this line, assuming a prevalence of ACN of 20% (8% CRC, 12% AA) [25], a minimum sample size cohort of 600 individuals was required (expected events of 120). During the timeline of the study (March-2014 to May-2015) we could derive a larger cohort of *N* = 761 individuals in order to guarantee the number of dummy variables. From these individuals FIT was not available in 30 out of them, leading to a final cohort of *N* = 731 individuals.

**Fig. 1:**
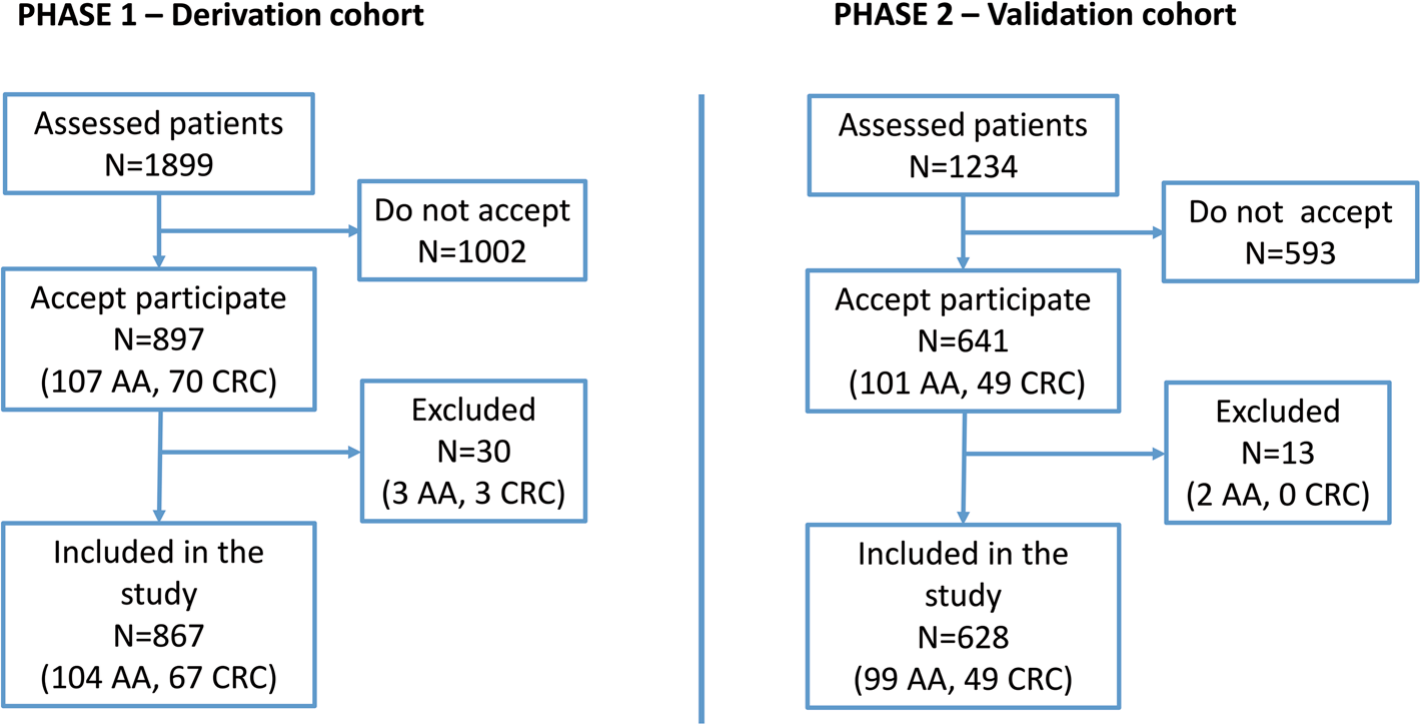
Diagram flow of participants through the study in both the derivation and validation cohorts, including the number of participants with the main outcome

We settled that a minimum sample size of 500 was required for the validation phase (see Additional file 3). Finally, we recruited a cohort of 527 individuals. In addition, symptomatic patients with no indication for a fast-track colonoscopy and negative FIT were included, to have enough patients with negative FIT in the low-risk group (finally, 136 in phase 1 and 101 in phase 2). This totals 867 individuals in phase 1 (731 + 136) with 171 events (104 advanced adenomas and 67 colorectal cancers), and 628 individuals in phase 2 (527 + 101) with 148 events (99 AA and 49 CRC) (Fig. 1).

### Derivation of the FIT variables included in the model

Three samples for FIT were obtained in each of the *N* = 867 individuals and we used the maximum f-Hb value (MAXFIT) out of the three samples in each individual. Since the variable f-Hb did not follow a Normal distribution even after logarithmic transformation, as has been reported elsewhere [17], and the risk of CRC did not have a linear relationship to f-Hb, MAXFIT was introduced as a categorical variable. We selected as cut-off 11 μg Hb/g faeces (see Additional file 3). Therefore, we assumed three categories for the MAXFIT variable: ≤4, > 4 to 11, > 11 μg Hb/g faeces. In addition, since we collected three faecal samples, we also used a variable which counts the number of samples with FIT> 4 μg Hb/g faeces (NSAMPLES> 4). This variable could take values in the range from 0 to 3 and was introduced in the model as an interaction term with MAXFIT variable.

### Development of the risk score

Univariate analysis was carried out on the derivation set using the Pearson chi-square method to examine the association between clinical risk factors and advanced neoplasia. In our study, age and MAXFIT were the variables associated with higher ORs in the univariate analyses (see Tables 1 and 2), and it is in accordance with results reported in previous studies [17]. Unlike other studies, we have also considered the number of positive samples, since this variable improved the predictive performance of the model. Thus, the prognostic variables included in the seminal multivariate logistic model were Age, MAXFIT and NSAMPLES> 4. Then, significant variables in the univariate analysis (*p* < 0.05) were introduced one by one into the seminal multivariate model and its C-Statistic and Brier score were recorded. Those variables that significantly improved the performance indexes (higher C-Statistic and lower Brier score) compared against the seminal values were chosen to be included in the final prognostic model (see Table 4). For each risk factor, we assigned a weight in the risk score using the respective odds ratio (OR) yielded by the logistic regression, where the maximum log-OR received a score of 10 points. The risk score for an individual was the sum of their individual risk factors. Risk groups were classified according to the ‘*AddFor’* algorithm, which allows for categorization of continuous variables in prediction models within a logistic regression model, in such a way that the best discriminative ability is obtained in terms of the highest C-Statistic [26]. This methodology allows for the selection of more than one cut point and better risk classification of patients.

**Table 1:** Crude and adjusted ORs of age and FIT variables for association with ACN in the derivation (Phase I) cohort (Seminal model: C-Statistic = 0.846; Brier Score = 0.115; Hosmer-Lemeshow *p*-value = 0.796)

X	Control group (non-AA, non-CRC)	AA+CRC	OR (Crude)	OR (Adjusted)
Age group (years)				
≤ 40	66 (9.48%)	1 (0.58%)	Ref.	Ref.
40–50	98 (14.1%)	6 (3.51%)	3.61 [0.58;94.9]	4.62 [0.49;43.6]
50–60	157 (22.6%)	34 (19.9%)	12.5 [2.63;299]	17.9 [2.25;142.4]
> 60	375 (53.9%)	130 (76.0%)	20.0 [4.42;469]	21.54 [2.8;165.6]
MAXFIT (out of 3 samples)				
≤ 11	508 (73.0%)	33 (19.3%)	Ref.	Ref.
> 11	188 (27.0%)	138 (80.7%)	11.2 [7.49;17.3]	2.31 [0.99;5.38]
NSAMPLES> 4				
0	441 (63.4%)	24 (14.0%)	Ref.	Ref.
1	110 (15.8%)	18 (10.5%)	3.01 [1.55;5.74]	2.98 [1.54;5.8]
2	71 (10.2%)	18 (10.5%)	4.65 [2.37;9.01]	4.49 [2.3;8.8]
3	74 (10.6%)	111 (64.9%)	27.2 [16.7;46.0]	28.1 [16.8;46.9]

MAXFIT: maximum f-Hb value; NSAMPLES > 4: Number of samples with FIT > 4 μg Hb/g faeces

**Table 2:** Univariate and age- and FIT-adjusted predictors of ACN in the derivation (Phase I) cohort: Crude and adjusted ORs, and C-Statistic and Brier score of the adjusted model

VARIABLE	Control group (Non-AA, non-CRC)	AA+CRC	OR (Crude) [95%CI]	OR (Adjusted) [95%CI]	C-Statistic/ Brier score adjusted model
	*N* = 696	*N* = 171			
Gender					
Man	311 (44.7%)	103 (60.2%)	Ref.	Ref.	
Woman	385 (55.3%)	68 (39.8%)	0.53 [0.38;0.75]	0.81 [0.53;1.21]	0.841 / 0.1158
Body Mass Index (Kg/m^2^)					
≤ 25	246 (35.3%)	47 (27.5%)	Ref.	Ref.	
> 25	450 (64.7%)	124 (72.5%)	1.44 [1.01;2.10]	1.19 [0.77;1.85]	0.841 / 0.1161
Colonoscopy (5 years before)					
No	555 (79.7%)	153 (89.5%)	Ref.	Ref.	
Yes	141 (20.3%)	18 (10.5%)	0.47 [0.27;0.77]	0.35 [0.21;0.63]	0.862 / 0.1031
Smoking (History)					
No	359 (51.6%)	77 (45.0%)	Ref.	Ref.	
Yes (Ex/Current)	337 (48.4%)	94 (55.0%)	1.30 [0.93;1.82]	1.51 [1.02;2.29]	0.859 / 0.1050
Smoking (Years):					
No	359 (51.6%)	77 (45.0%)	Ref.	Ref.	
≤ 42	191 (27.4%)	25 (14.6%)	0.61 [0.37;0.98]	1.17 [0.6;2.33]	
> 42	146 (21.0%)	69 (40.4%)	2.20 [1.51;3.21]	1.65 [0.99;2.61]	0.849 / 0.1086
Drugs (NSAIDs or antiplatelet or anticoagulants)					
No	415 (59.6%)	98 (57.3%)	Ref.	Ref.	
Yes	281 (40.4%)	73 (42.7%)	1.10 [0.78;1.54]	0.79 [0.52;1.21]	0.841 / 0.1153
Anticoagulants					
No	673 (96.7%)	156 (91.2%)	Ref.	Ref.	
Yes	23 (3.30%)	15 (8.77%)	2.82 [1.40;5.50]	1.67 [0.75;3.80]	0.842 / 0.1159
NSAIDs					
No	525 (75.4%)	136 (79.5%)	Ref.	Ref.	
Yes	171 (24.6%)	35 (20.5%)	0.79 [0.52;1.18]	0.79 [0.49;1.29]	0.846 / 0.1156
Abdominal pain					
No	299 (43.0%)	97 (56.7%)	Ref.	Ref.	
Yes	397 (57.0%)	74 (43.3%)	0.58 [0.41;0.81]	0.77 [0.52;1.16]	0.840 / 0.116
Iron deficiency					
No	237 (34.1%)	56 (32.7%)	Ref.	Ref.	
Yes	174 (25.0%)	55 (32.2%)	1.34 [0.88;2.04]	0.92 [0.55;1.92]	
Unknown	285 (40.9%)	60 (35.1%)	0.89 [0.59;1.34]	0.86 [0.54;1.37]	0.840 / 0.116
Iron deficiency anaemia					
No	371 (53.3%)	77 (45.0%)	Ref.	Ref.	
Yes	166 (23.9%)	54 (31.6%)	1.57 [1.05;2.32]	1.13 [0.69;1.84]	
Unknown	159 (22.8%)	40 (23.4%)	1.21 [0.79;1.85]	1.14 [0.69;1.91]	0.840 / 0.116
Type change bowel habit					
No	261 (37.5%)	78 (45.6%)	Ref.	Ref.	
Diarrhoea	226 (32.5%)	59 (34.5%)	0.87 [0.59;1.28]	1.08 [0.68;1.71]	
Constipation	179 (25.7%)	31 (18.1%)	0.58 [0.36;0.91]	0.71 [0.42;1.22]	
Unknown	30 (4.31%)	3 (1.75%)	0.35 [0.08;1.02]	0.78 [0.23;2.74]	0.840 / 0.1153
Abdominal pain duration					
No	299 (43.0%)	97 (56.7%)	Ref.	Ref.	
Yes, 1–12 Days	123 (17.7%)	26 (15.2%)	0.65 [0.40;1.05]	0.65 [0.37;1.15]	
Yes, > 12 Days	274 (39.4%)	48 (28.1%)	0.54 [0.37;0.79]	0.86 [0.54;1.37]	0.850 / 0.1157
Rectal bleeding pattern					
No	342 (49.1%)	84 (49.1%)	Ref.	Ref.	
Yes, without specific pattern	219 (31.5%)	43 (25.1%)	0.80 [0.53;1.20]	0.59 [0.36;1.07]	
Yes, with specific pattern	135 (19.4%)	44 (25.7%)	1.44 [0.97;2.12]	1.33 [0.87;2.01]	0.849 / 0.1151
Rectal bleeding duration (days)					
No rectal bleeding	342 (49.1%)	84 (49.1%)	Ref.	Ref.	
≤ 30	328 (47.1%)	74 (43.3%)	0.92 [0.65;1.30]	0.63 [0.41;1.05]	
> 30	26 (3.74%)	13 (7.60%)	2.04 [0.97;4.10]	0.86 [0.37;1.98]	0.846 / 0.1150
Haemoglobin (g/dL)					
≤ 11	105 (15.1%)	43 (25.1%)	Ref.	Ref.	
> 11	481 (69.1%)	112 (65.5%)	0.57 [0.38;0.86]	1.02 [0.61;1.71]	
Unknown	110 (15.8%)	16 (9.36%)	0.36 [0.18;0.67]	0.62 [0.31;1.31]	0.847 / 0.1127
Ferritin (ng/mL)					
≤ 40	176 (25.3%)	56 (32.7%)	Ref.	Ref.	
> 40	225 (32.3%)	53 (31.0%)	0.74 [0.48;1.13]	0.82 [0.51;1.34]	
Unknown	295 (42.4%)	62 (36.3%)	0.66 [0.44;0.99]	0.85 [0.52;1.39]	0.849 / 0.1106
CEA (ng/mL)					
≤ 12	610 (87.6%)	135 (78.9%)	Ref.	Ref.	
> 12	5 (0.72%)	11 (6.43%)	9.73 [3.43;32.1]	2.91 [0.88;9.36]	
Unknown	81 (11.6%)	25 (14.6%)	1.40 [0.85;2.25]	1.27 [0.71;2.27]	0.849 / 0.1101

### Statistical analysis

The development and external validation of a multivariable prediction model study was designed according to TRIPOD statement [27]. A checklist indicating the pages where information for each item is reported can be found as a supplementary file (Additional file 4).

Statistical analysis was performed with R software [28]. Variables considered after variable selection in univariate analysis are listed in Tables 1 and 2. Other analysed variables such as family or personal history of CRC or colonic polyps were no informative. Missing-data were introduced as ‘Unknown’ and analysed as a new category of each variable; this was mainly observed for analytical variables related to iron deficiency since at inclusion many patients had received oral iron supplements.

A Bayesian logistic regression model accounting for age and NSAMPLES> 4 depending on the levels of MAXFIT was fitted to each one of the variables considered, assuming a non-informative Cauchy prior for the model parameters [29]. Once the model was fitted, we derived the median OR and their 95% credible interval from the subsequent distribution of the model parameters. The C-Statistic and the Brier score were used as the overall performance measures of the modelling [30], selecting the model with maximum C-Statistic and minimum Brier score. Finally, the Hosmer-Lemeshow test was used to test the calibration of the model in the validation cohort [24, 30].

To assess the effect of using 3-sample FIT as compared to 1-sample FIT, we developed different models created with a FIT value randomly chosen from the 3 values of each patient. Also we compared the 3-sample FIT final model with a previously published 1-sample FIT test score (FAST score) [17]. For these comparisons we used the method proposed by DeLong et al. [31], as implemented by Robin et al. in pROC package [32].

## Results

### Description of the derivation and validation cohorts

Between April 2014 and February 2017, 1538 patients were included, 897 in the derivation (phase 1) and 641 in the validation (phase 2) cohorts (Fig. 1). Another 1595 patients either did not agree to participate or were not contacted with enough time before the colonoscopy. There were no differences in demographic characteristics and indications for colonoscopy between these and the included patients (data not shown). Forty-three patients (2.8%) were excluded because of improper FIT collection. The indications for colonoscopy and the baseline characteristics of the patients included in each phase are shown in Additional file 6 and Additional file 7. One thousand fifty-eight patients met the NICE criteria for a fast-track colonoscopy and 237 did not (136 in phase 1 and 101 in phase 2).

We detected CRC in 67/867 (7.7%) in study phase 1, and 49/628 (7.8%) in phase 2. No patient of the subgroup with negative FIT and who did not met the NICE criteria for a fast-track colonoscopy had a CRC. There were no significant differences in tumour localization and staging between the two phases (Additional file 5). Additionally, we found advanced adenomas in 203 patients, 104 (11.9%) in phase 1 (5 with no NICE criteria and negative FIT) and 99 (15.7%) in phase 2 (6 with no NICE criteria and negative FIT). Six adenomas in phase 1 had intramucosal carcinoma (Tis).

There were no significant differences in demographic and clinical variables between phase 1 and phase 2 included patients (Additional file 7). However, there were significant differences in the FIT variables (MAXFIT and NSAMPLES> 4). In fact, all CRC patients in phase 1 had positive FIT with values above 11 μg Hb/g faeces. In contrast, there were three CRC patients in phase 2 with negative FIT, and two patients with values > 4 and < 11 μg Hb/g faeces. Overall, there were 6/116 (5%) CRC patients with only 1 out of 3 faecal samples positive.

### Derivation of the predictive risk score

Age and FIT variables were independently associated with the risk of ACN, with ORs very much higher than other variables (Table 1). Univariate and age- and FIT-adjusted predictors of ACN in the derivation (Phase I) cohort are presented in Table 2. Only ‘Colonoscopy up to 5 years before FIT’, ‘smoking history’, and ‘smoking years’ retained overall statistical significance and improve C-Statistic and Brier score of the multivariate analyses. Both in the derivation and the validation cohorts, the C-Statistic with ‘smoking history’ was slightly better; thus we selected this simpler variable for the derived score. The derived predictive risk score is described in Table 3, which shows the multivariate predictors of ACN using the model fitted to the derivation (Phase I) cohort. The predictive score ranged from − 4 to 24 points. The predictive performance of the final risk score for ACN was excellent with a C-Statistic of 0.865 (95% CI, 0.83–0.89). Taking into account only CRC, the C-Statistic was 0.93 (95% CI, 0.91–0.95).

**Table 3:** Multivariate predictors of ACN using the model fitted to the derivation (Phase I) cohort. The predictive score derived ranged from −4 to 24 points (Final model: C-Statistic = 0.865; Brier Score = 0.10; Hosmer-Lemeshow p-value = 0.86)

Risk Factor	OR	Q 2.5	Q 97.5	Points
Age 40–50 years	2.26	0.52	10.05	3
Age 50–60 years	7.58	2.02	28.56	8
Age > 60 years	11.88	3.27	43.13	10
Colonoscopy (in the previous 5 years) (Yes)	0.36	0.2	0.65	−4
Smoking (Yes)	1.49	1.01	2.27	2
*IF MAXFIT [> 4–11]*				
NSAMPLES> 4	1.72	1.04	2.83	2x†
*IF MAXFIT > 11*				
NSAMPLES> 4	2.89	2.45	3.44	4x†

MAXFIT: maximum f-Hb value; NSAMPLES > 4: Number of samples with FIT > 4 μg Hb/g faeces. Discrete variable (0 to 3). Points: Points assigned to each Risk Factor

†Note on 2x/4x: factor to multiply NSAMPLES

How do we interpret the model? Ex: Suppose a 55-year-old man has not smoked or has done a prior colonoscopy and with values of FIT 4, 6 and 10 μg Hb/g faeces. This individual has a MAXFIT value of 10, and 2 samples with FIT > 4 (2 positive samples); therefore its score will be 8 points by age + 2 points per FIT (corresponding to the OR = 1.72) multiplied by 2 positive samples, giving 4 points. Total: 12 points. But if their FIT values were 4, 8, and 12 μg Hb/g faeces, the MAXFIT value of 12 and two positive samples, their score would be 8 points by age + 4 points per FIT (corresponding to the OR = 2.89) multiplied by 2 positive samples, giving 8 points. Total: 16 points

### Effect of the number of positive samples on the predictive performance of the model

We compared the model resulting from taking 1 random sample out of the 3 samples with the proposed final model for ACN which includes MAXFIT and NSAMPLES (Table 4). This was done 5 times. The C-Statistic from the five random samples were lower than that of the proposed final model, showing that the final model including the interaction between MAXFIT and NSAMPLES classified better a 2.2–3% of patients.

**Table 4:** Comparison of the model resulting from taking 1 random sample out of the 3 FIT samples with the proposed final model for ACN

MODEL	AUC	CI 95% (Bootstrap)
With interaction^†^	0.865	0.833–0.894
RANDOM – 1^‡^	0.844	0.809–0.875
RANDOM – 2	0.835	0.800–0.897
RANDOM – 3	0.843	0.809–0.876
RANDOM – 4	0.842	0.808–0.875
RANDOM – 5	0.842	0.807–0.875

^†^Final model including MAXFIT and NSAMPLES

^‡^RANDOM-N: Model created with a FIT value randomly chosen from the 3 values ​​of each patient. This was repeated 5 times

MAXFIT: maximum f-Hb value; NSAMPLES> 4: Number of samples with FIT > 4 μg Hb/g faeces

**Fig. 2:**
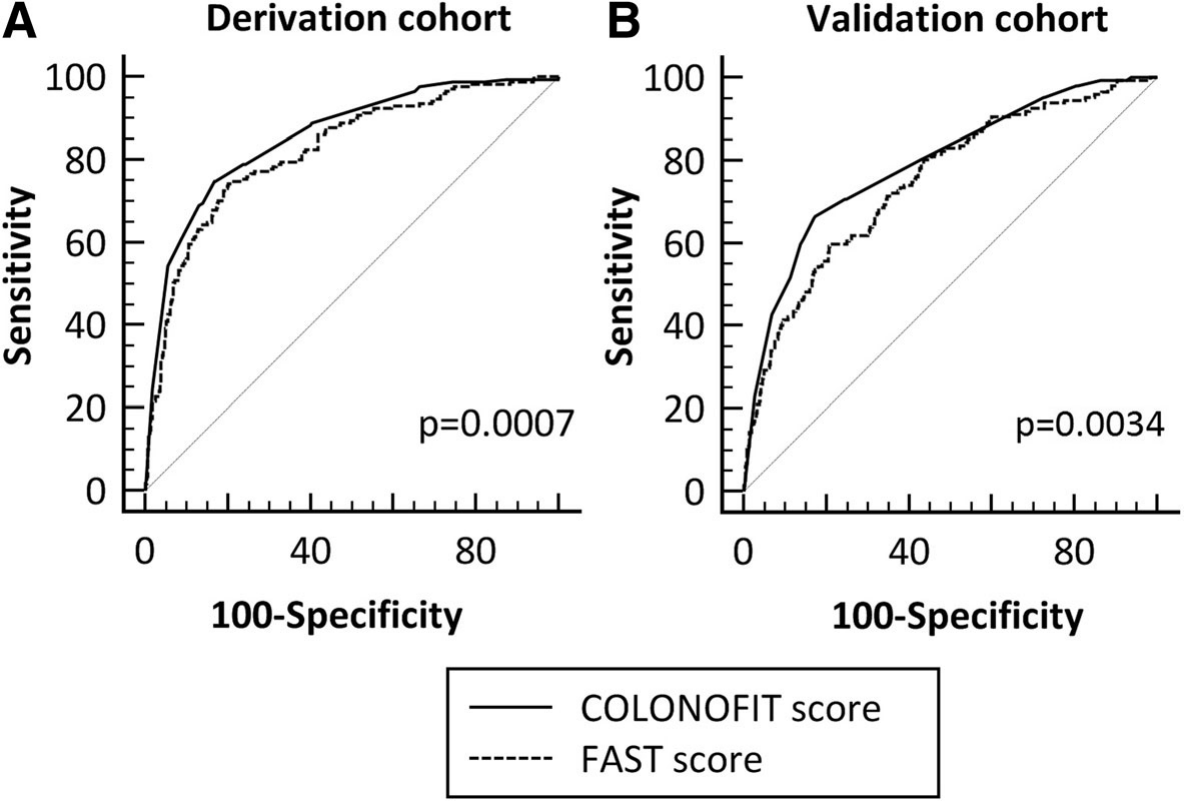
Comparison of the C-Statistic of the present 3-sample FIT model (COLONOFIT score) with the 1-sample FIT, age and sex test (FAST) score^17^ for ACN diagnosis in both the derivation and validation phase

In addition, we compared our 3-sample FIT final model (COLONOFIT score) with the 1-sample FIT, age and sex test (FAST) score developed by Cubiella et al. [17] In study phase 1, the C-Statistic for both CRC and ACN was significantly higher with COLONOFIT than FAST score (CRC, 0.93 ± 0.009 vs. 0.90 ± 0.01; *p* = 0.04; and ACN, 0.86 ± 0.02 vs. 0.82 ± 0.02; *p* = 0.0007) (Fig. 2). In addition, in study phase 2, the C-Statistic for both CRC and ACN was also higher with COLONOFIT than FAST score (CRC, 0.86 ± 0.025 vs. 0.83 ± 0.03; *p* = 0.18; and ACN, 0.79 ± 0.02 vs. 0.75 ± 0.02; *p* = 0.0034). The differences between both scores were maintained after excluding the patients who did not met NICE criteria and had negative FIT (data not shown).

### Reliability of the model in the validation cohort

As above mentioned, the risk-score was more accurate for ACN and CRC detection in the derivation than in the validation cohort. However, its performance remained good. The Hosmer-Lemeshow *p*-value was 0.86, showing that the model was well calibrated.

Patients of the validation cohort were categorized into risk subgroups according the presence of ACN and the value of the final risk-score using the *‘AddFor’* algorithm (see methods) (Table 5). The probability (or prevalence) of ACN for a risk-score > 20 points was 66%, whereas for a risk-score ≤ 10 points it was 10%. If we take into account only CRCs, these probabilities were 32 and 1%, respectively.

**Table 5:** Probability of an individual classified based on its risk score (after applying the score derived from Phase 1 to the validation cohort, Phase 2). The cut-off points were selected using the *‘AddFor’* algorithm (see methods)

Score	Prob (Control group)*	Prob (AA)	Prob (CRC)	Prob (Control group)* (95% CI)	Prob (AA+CRC) (95% CI)
> 20	0.34	0.34	0.32	0.34 (0.28; 0.39)	0.66 (0.51; 0.76)
10–20	0.76	0.16	0.07	0.76 (0.65; 0.86)	0.24 (0.14; 0.31)
≤10	0.90	0.09	0.01	0.90 (0.84; 0.97)	0.10 (0.01; 0.16)

*Control group: Non-AA, non-CCR

A risk-score > 10 points allowed us to diagnose 96% of CRC and 72% of AA, with only needing to prioritize a 50% of colonoscopies (Table 6). No patient with a risk-score < 4 points had CRC but in this case we need to prioritize 95% of colonoscopies. Higher cut-offs would prioritize fewer colonoscopies while increasing the percentage of CRC loss.

**Table 6:** Accuracy of a positive risk score (> 10 points) to diagnose both ACN and CCR in the validation cohort (Phase II) and in the derivation *plus* validation cohorts (Phase I + Phase II). Frequency of colonoscopy prioritization with the associated miss of either CRC or AA cases for a Score > 10

PHASE II			
	ACN	Control group (non-AA, non-CRC)	Total
Score > 10	118 (47 CCR)	198	316
Score ≤ 10	30 (2 CCR)	282	312
Total	148	480	628
	ACN		CRC
Sensitivity (%)	79 (72–85.4)^†^		96 (85–99)
Specificity (%)	58 (54.2–63)		52 (48–56)
PPV (%)	37 (32–42.7)		14.4 (11–19)
NPV (%)	90 (87–93.2)		99.3 (97–99.9)
Diagnostic OR	5.6 (3.6–8.7)		25.4 (6.1–106)
LR (+)	1.93 (1.7–2.2)		1.99 (1.8–2.2)
LR (−)	0.34 (0.25–0.48)		0.08 (0.02–0.30)
Prevalence (%)	23.6		7.8
*Score ≤ 10*	N	(%) Loss	
Missed cases of CRC	2	4.1	
Missed cases of AA	28	28.3	
Score > 10	N	(%) Prioritization	
Prioritization	316	50.3	
PHASE I + PHASE II			
	ACN	Control group (non-AA, non-CRC	Total
Score > 10	270 (114 CRC)	468	738
Score ≤ 10	49 (2 CRC)	708	757
Total	319	1176	1495
	ACN		CRC
Sensitivity (%)	85 (80.3–88)^†^		98 (93–99.7)
Specificity (%)	60 (57.4–63)		53 (51–56)
PPV	36 (33.2–40)		15 (13–18)
NPV	93.5 (91.5–95)		99.7 (99–100)
Diagnostic OR	8.3 (6–11.5)		65.2 (16–265)
LR (+)	2.12 (1.95–2.31)		2.10 (1.98–2.24)
LR (−)	0.25 (0.20–0.33)		0.03 (0.008–0.13)
Prevalence (%)	21.3		7.76
*Score ≤ 10*	N	(%) Loss	
Missed cases of CRC	2	1.7	
Missed cases of AA	47	23	
Score > 10	N	(%) Prioritization	
Prioritization	738	49.3	

^†^95% CI

## Discussion

A multivariable prediction modelling study to assess the pre-test-probability of ACN in symptomatic patients with indication of a fast-track colonoscopy was performed. This approach has been considered the most efficient method of capturing the effects of clinical judgement [33]. The derived and validated risk-score (COLONOFIT score) may be useful to prioritize colonoscopies in patients fulfilling criteria for a fast-track exploration. We saw as more relevant both the percentage of missed cases of CRC and the percentage of patients to prioritize than sensitivity and specificity to detect ACN. In fact, a risk-score > 10 points, which would imply to prioritize 50% of eligible patients, allowed the overall diagnosis of 98% of CRC (2% of missed CRC cases) and 77% of AA. It has been shown that decreasing the positivity threshold of FIT does not increase the detection rate of ACN, and thus it is assumed that not all CRCs can be detected by using FIT [34, 35].

A 3-fecal sample FIT regime was used in order to increase the CRC detection rate, selecting the cut-off to consider a sample as positive on the basis of the DOR. This cut-off was lower than that used in the screening program of average-risk asymptomatic patients in our geographical area. The logistic regression analysis showed that in addition to age, a maximum f-Hb level > 11 μg Hb/g faeces and the number of positive samples (> 4 μg Hb/g faeces) were the variables associated with the highest ORs to predict ACN. Noteworthy, having three positive samples was associated with ACN with the highest OR and C-Statistic. In contrast, no clinical symptom or clinical presentation was independently associated with the risk of ACN after adjusting for age and FIT variables. In fact, the low predictive value of clinical symptoms has been previously reported [8, 10, 33]. In addition to age and FIT variables, smoking increased while a previous colonoscopy (in the last 5 years) decreased the risk.

Some previous studies had suggested the use of FIT with other clinical variables or even only one-sample FIT *plus* age and sex (FAST score) as a predictive tool of CRC in symptomatic patients, showing more accuracy than symptom-based referral criteria [11, 17]. NICE referral criteria for suspected CRC have a 68.2% sensitivity and a 50.2% specificity for CRC detection and, actually, these criteria cannot rule out CRC [11]. In the present study, we showed that a COLONOTIF score > 10 points has a sensitivity of 98% and a specificity of 53% to detect CRC, and in this sense the NPV was 99.7% and the LR- 0.03. Although the specificity was not very high and a low specificity is associated with unnecessary referrals, it should be emphasized that a score > 10 allowed to prioritize 50% of patients fulfilling NICE referral criteria, detecting 98% of CRCs, and avoiding 50% of fast-track colonoscopies. Furthermore, 70% of patients with CRC had a score > 20 points, which was associated with a specificity of 90%. The present comparison shows that COLONOFIT score classified patients a 3–4% better than the FAST score in both the derivation and validation cohorts. Further studies on a direct comparison of both scores are needed to assess if the 3–4% gain in classification could be offset by lower adherence (by submitting 3 vs. 1 FIT).

Selecting patients attending primary care may prevent a selection bias of patients with higher prior probability of CRC [36]. The present study included mainly patients referred by primary care for a fast-track colonoscopy and, anyhow, the variable primary or secondary care referral was included in the analysis without showing significant association with ACN. Thus, the derived risk-score may guide primary care physicians in their referral decisions for a fast-track colonoscopy. COLONOFIT score allows risk-stratifying of patients in clinical practice to inform decision-making. Those patients with a score > 20 (15% of the patients in the validation cohort) had a risk of ACN of 66% (32% CRC, 34% AA) and should be sent for a 2WW colonoscopy. In contrast, those with a score ≤ 10 (50% of patients in the validation cohort) had a risk of ACN of 10% (1% CRC, 9% AA), and should be referred to either gastroenterology or other appropriate clinic in secondary care or a colonoscopy in the conventional slower referral route.

After developing the prediction model in the derivation cohort, its performance was evaluated in a different cohort (validation cohort), collected using the same protocol and outcome definitions and measurements, but sampled from a later period. Such external validation implies that for each individual in the new data set, outcome predictions were made using the derived predictive score [37, 38]. The performance of a predictive model is typically worse when evaluated on samples independent of the sample used to develop the model. In fact, in phase 1 of the study all CRC patients and 82% of AA had a score > 10; however, the performance characteristics in the validation cohort remained good.

Prevalence of CRC in symptomatic patients of the present study was near 8%, and that of ACN around 24%, which are figures much higher than those observed in the CRC screening populations (typically, ACN has a prevalence < 10% in that setting), and similar to other previous studies [11, 17]. This reflects the need for a different diagnostic approach in the two settings, and in this sense, lower cut-offs of FIT have been suggested for symptomatic patients [7]. Results of the present study suggest that using 3-sample FIT (on three different days) is an additional diagnostic strategy that increases the prior probability of ACN. The 3-sampling strategy for FOBT was traditionally used for guaiac-based tests, and was abandoned when introducing the more sensitive FITs, and a meta-analysis showed that in average-risk asymptomatic patients increasing the number of FIT samples did not affect the pooled performance characteristics of FITs for CRC [18]. It was concluded that a 1-sample FIT regimen for CRC detection might ultimately be desirable, given the importance of optimizing overall adherence in repeated rounds of biennial testing for programmatic screening. However, several studies have directly evaluated the effect of FIT sample number on the diagnostic accuracy of FITs in average-risk asymptomatic participants [19–21], and in symptomatic patients [22, 23], suggesting that using either 2 or 3 tests provided the best discrimination for CRC. Therefore, these data and the results of the present study suggest that using 3-sample FIT may increase the detection rate of ACN in patients with risk symptoms to an acceptable number needed to scope.

Not all studies on statistical risk models to predict CRC in people with symptoms included AA in addition to CRC. However, it is widely accepted that CRC arises from the adenoma-carcinoma sequence and so identification of patients with high-risk adenoma has the potential to reduce future incidence of invasive CRC and prevent mortality. A systematic review performed in 2016 included only two models assessing CRC *plus* AA [13], both of which reported only limited performance data and which have not been externally validated [39, 40]. We think that the priority of the model should be to identify both prevalent CRC and the patients at high risk of developing CRC in the future, and in this sense, the COLONOFIT score prioritizes symptomatic patients to detect both CRC and AA.

This study has specific strengths that deserve to be highlighted. All included patients had a colonoscopy (even those with negative FIT); the best cut-off point of FIT was selected on the basis of the results, using the DOR value which maximizes the probability of ACN; a 3-sample FIT regime was evaluated to assess whether the number of positive samples influenced the probability of ACN; a face-to-face structured and detailed survey was prospectively performed by a trained person; the external validation of the score was performed in a new cohort selected later in time; the derived risk score is ease of use, and all its components are readily available to general practitioners. The study has also some limitations. First, not all included patients were primary care referrals, but as noted earlier, there was no significant association with the referral origin, and all physicians used the same criteria to refer a patient for a fast-track colonoscopy that were reviewed by the case manager before inclusion. Second, the included patients fulfilled the criteria for a fast-track colonoscopy; thus the use of the predictive score cannot be generalized to patients without these criteria without further studies. Finally, there were missing data about iron status (patients were receiving iron supplements) without statistical significance comparing the two study phases, which could have precluded finding a significant association.

## Conclusion

A risk-scoring system was derived and validated to prioritize fast-track colonoscopies, which was shown to be efficient, simple, and robust. Further external validation studies are needed to warrant the widespread recommendation of the model in clinical practice.

## Additional files

Additional file 1: Study protocol. (DOCX 201 kb)

Additional file 2: FIT performance. (DOCX 12 kb)

Additional file 3: Supplementary statistical methods. (DOCX 12 kb)

Additional file 4: Tripod checklist. (DOCX 16 kb)

Additional file 5: **Figure S1.** Tumour localization and staging in both study phases. (DOCX 11 kb)

Additional file 6: **Table S1** Indication for colonoscopy in both study phases. (DOCX 12 kb)

Additional file 7: **Table S2** Description of patients included in study phase 1 and 2. (DOCX 22 kb)

Additional file 8: Dataset supporting the conclusions of this article. (ZIP 1179 kb)

## Data Availability

Data supporting the findings of this paper are available as Supplementary information (Additional file 8).

## Abbreviations

2WW: 2-week wait
AA: Advanced adenoma
ACN: Advanced colonic neoplasia
CRC: Colorectal cancer
f-Hb: Faecal haemoglobin
FIT: Faecal immunological occult haemoglobin test
MAXFIT: Maximum f-Hb value
