# Supplementary material for: Prediction of advanced colonic neoplasm in symptomatic patients: a scoring system to prioritize colonoscopy (COLONOFIT study)
Source: BMC Cancer. 2019 Jul 25;19:734. doi: 10.1186/s12885-019-5926-4 (PMC6659265; doi:10.1186/s12885-019-5926-4)
Supplement: Supplementary file 1 — Study protocol. (DOCX 201 kb) [file 12885_2019_5926_MOESM1_ESM.docx]

**2. BACKGROUND AND STATE OF THE ART, INCLUDING RELEVANT BIBLIOGRAPHY**

Colorectal cancer (CRC) is the third most common cancer in men and the second in women worldwide. In Spain 25,000 new cases are diagnosed each year and around 13,000 deaths occur from this neoplasm (1). Despite progress made in recent years in the diagnosis and treatment of this disease, a significant improvement in survival at 5 years has not been shown, which persists around 50%. This is because over 80% of new cases are symptomatic patients, and the disease is advanced at the time of diagnosis. Most cases of CRC develop from advanced adenomas (>1 cm, high-grade dysplasia or villous component), which if detected could endoscopically be removed, thus preventing CRC developing. Then, CRC is a preventable disease.

A significant percentage of CRC patients are diagnosed based on the presence of clinical symptoms associated with this malignancy (2-4). It is therefore important to identify patients who have symptoms and/or signs of suspicion, so an early colonoscopy could be indicated. The most common signs and symptoms associated with CRC are rectal bleeding and change of bowel movements. Iron deficiency anaemia can also be a presentation of CCR. However, these clinical symptoms are non-specific, since they are also observed in other GI diseases. The positive predictive value (PPV) of rectal bleeding for colorectal adenomas was estimated to range from 3 to 8% in general population, 7-12% in primary care consultations, and 8-32% in specialized care. The PPV for CRC is less than 1% in the general population, ranges from 2 to 15% in primary care consultations, and reaches 40% in specialized care (5-8). The presence of signs and symptoms associated with CRC may modify the PPV (7,8). The diagnostic suspicion of CRC increases with age, male sex, the change in the bowel movements and the blood mixed with the stool (as opposed to the blood covering the stool). The dark colour of blood and mucous mixed with blood in the stool is also associated with a higher risk of CRC. In contrast, other studies have observed that patients with rectal bleeding associated with perianal symptoms have a very low probability of CRC, especially when they are not associated with changes in the bowel movements, and particularly if a rectal mass has been excluded. Also, presence of an abdominal mass, occlusive abdominal pain, weight loss, and/or constitutional syndrome may be indicative of a CRC.

A short elapsed time between the CRC suspicion and the diagnosis and treatment of the disease is desirable. A long elapsed time may be due to a delay attributable to either the health services or the patient himself, who in most cases does not give relevance to the symptoms and has fear of cancer diagnosis. Assistance in different areas may be helpful to reduce the diagnostic delay. Recently, the proposed rule of 2 week wait (2WW) referral system for the NHS (UK) has been revaluated. This system was proposed in 2000 to ensure that specialists in reference hospitals assess all patients with suspected CRC within 14 days after the urgent referral by a primary care physician (11). Despite that 77% of patients with CRC were referred by primary care physicians via urgent pathways, this system did not improve 5-year colorectal cancer survival (12). In addition, a systematic review indicated that there was no association between delayed diagnosis and treatment, and survival of patients with CRC (9,10). This finding may be due to that symptoms when present are very often indicating an advanced disease, and once a cancer became symptomatic, early treatment did not improve survival. In this sense, it has been shown that there was no evidence of stage migration following the introduction of the 2WW referral system, and an association between delay in diagnosis and stage of disease has not been demonstrated (9,10,11,13).

However, introduction of a fast-track service to meet the 2WW target resulted in a trend towards fewer emergency presentations with CRC (14). In addition, regardless of the effect on prognosis, late diagnosis creates uncertainty and anxiety in the patient and, therefore, the current health plans prioritize the rapid diagnosis of cancer (15). The 2WW rule has been criticized because of the low overall cancer detection rate due to the poor specificity of the patient clinical symptoms resulting in overwhelming referral rates. The present limited resources have worsened the situation. The other significant criticism of the 2WW rule has been its failure to detect a significant proportion of patients with CRC who present through the conventional slower referral route.

On the other hand, there is conclusive evidence that screening in the average-risk population for CRC by faecal occult blood test (FOBT) improves survival significantly (16). Polyps and early CRC are characterized by negligible loss of blood in the stool intermittently, which can be detected by FOBT. Immunological methods whose target molecule is human hemoglobin do not require dietary restrictions in the days before and during the conduct of the tests. In a systematic review on the diagnostic validity of FOBTs, sensitivities for the detection of all neoplasms ranged from 6.2% (specificity 98.0%) to 83.3% (specificity 98.4%) for guaiac FOBTs, and 5.4% (specificity 98.5%) to 62.6% (specificity 94.3%) for immunochemical FOBTs. Specificity ranged from 65.0% (sensitivity 44.1%) to 99.0% (sensitivity 19.3%) for guaiac FOBTs, and 89.4% (sensitivity 30.3%) to 98.5% (sensitivity 5.4%) for immunochemical FOBTs. Sensitivities were higher for the detection of CRC, and lower for adenomas. There was no clear evidence to suggest whether guaiac or immunochemical FOBTs performed better, and poor reporting of data limited the scope of this review (17). A RCT comparing the classical guaiac FOBT (Hemoccult II®) with a fecal immunological test (FIT) (OC-Sensor®) in a population sample of 20,623 individuals aged 50-75 showed that FIT was significantly more effective than guaiac FOBT for the detection of CRC and advanced adenomas, but the specificity was lower (18). Also, a recent study performed in 2288 asymptomatic subjects 50-79 years of age in Spain showed that a sensitive FIT is markedly superior to the guaiac test for detecting significant colorectal neoplasia, and should be considered the first-choice FOBT for CRC screening in the average-risk population (19). However, the true FOBT accuracy in terms of sensitivity and specificity is difficult to find in the average-risk population because if the test is negative it would be difficult to perform a confirmatory colonoscopy. In contrast, if colonoscopy is indicated in patients with symptoms, it would be possible to determine the true sensitivity and specificity of these tests. However, in our knowledge that study has not been performed.

We suggested that FIT could be useful in prioritizing diagnostic colonoscopy in fast-track programs since this test could increase the sensitivity and specificity observed with patient clinical symptoms. Aiming to evaluate different ways to increase the specificity of the TIF we performed an study in 351 consecutive symptomatic patients referred for a diagnostic colonoscopy. The findings were that TIF with a low cut-off (50 mg/L buffer; 4 μg Hb/g stool) allowed to detect 9 out of 10 patients with advanced colonic neoplasia, with low specificity (27%). However, the TIF strategy with 3 samples per patient, allowed to increase the specificity to 75% when the 3 samples were positive. In addition, quantitative results increased the specificity until 75% when one of the 3 results was higher than 1500 mg/L, and even until 98%, when the sum of the three samples was over 5000 mg/L (20). This was a retrospective study and the impact of clinical symptoms in addition to the TIF to predict advanced colonic neoplasia could not be studied.

References

1. Castells et al. Guía clínica de prevención del cáncer colorrectal. Gastroenterol Hepatol 2009; 27:573-634.

2. Hamilton W, Round A, Sharp D, Peters TJ. Clinical features of colorectal cancer before diagnosis: a population-based case-control study. Br J Cancer. 2005;93:399-405.

3. Hamilton W, Sharp D. Diagnosis of colorectal cancer in primary care: the evidence base for guidelines. Fam Pract. 2004;21:99-106.

4. Lawrenson R, Logie J, Marks C. Risk of colorectal cancer in general practice patients presenting with rectal bleeding, change in bowel habit or anaemia. Eur J Cancer Care (Engl). 2006;15:267-71.

5. Jones R, Latinovic R, Charlton J, Gulliford MC. Alarm symptoms in early diagnosis of cancer in primary care: cohort study using General Practice Research Database. BMJ. 2007;334:1040.

6. Referral guidelines for suspected cancer. London: National Institute for Health and Clinical Excellence; 2005.

7. Selvachandran SN, Hodder RJ, Ballal MS, Jones P, Cade D. Prediction of colorectal cáncer by a patient consultation questionnaire and scoring system: a prospective study. Lancet. 2002;360:278-83.

8. Fijten GH, Starmans R, Muris JW, Schouten HJ, Blijham GH, Knottnerus JA. Predictive value of signs and symptoms for colorectal cancer in patients with rectal bleeding in general practice. Fam Pract. 1995;12:279-86.

9. Ramos M, Esteva M, Cabeza E, Campillo C, Llobera J, Aguilo A. Relationship of diagnostic and therapeutic delay with survival in colorectal cancer: a review. Eur J Cancer. 2007;43:2467-78.

10. Ramos M, Esteva M, Cabeza E, Llobera J, Ruiz A. Lack of association between diagnostic and therapeutic delay and stage of colorectal cancer. Eur J Cancer. 2008;44:510-21.

11. Thorne K, Hutchings HA, Elwyn G. The effects of the Two-Week Rule on NHS colorectal cancer diagnostic services: a systematic literature review. BMC Health Serv Res. 2006;6:43.

12. Zafar A, Mak T, Chapman MAS. The 2-week wait referral system does not improve 5-year colorectal cancer survival. Colorectal Dis 2011; 14:el77-el80.

13. Chohan DP, Goodwin K, Wilkinson S etal. How has the ‘two week wait’ rule affected the presentation of colorectal cancer? Colorectal Dis 2005; 7:450-3.

14. Davies RJ, Collins CD, Vickery CJ et al. Reduction in the proportion of patients with colorectal cancer presenting as an emergency following the introduction of fast-track flexible sigmoidoscopy : a three-year prospective study. Colorectal Dis 2004; 6:265-7.

15. Schneider C, Bevis PM, Durdey P et al. The association between referral source and outcome in patients with colorectal cancer. The Surgeon 2012, http://dx.doi.org/10.1016/j.surge.2012.10.004.

16. Hewitson P, Glasziou P, Irwig L, et al. Screening for colorectal cancer using the faecal occult blood test, Hemoccult. Cochrane Database Syst Rev 2007; 1:CD001216.

17. Burch JA, Soares-Weiser K, St John DJ, Duffy S, Smith S, Kleijnen J, et al. Diagnostic accuracy of faecal occult blood tests used in screening for colorectal cancer: a systematic review. J Med Screen. 2007;14:132-7.

18. Van Rossum LG, Van Rijn AF, Laheij RJ, Van Oijen MG, Fockens P, Van Krieken HH, et al. Random comparison of guaiac and immunochemical fecal occult blood tests for colorectal cancer in a screening population. Gastroenterology. 2008;135:82-90.

19. Parra-Blanco A, Gimeno-García AZ, Quintero E, et AL. Diagnostic accuracy of immunochemical versus guaiac faecal occult blood tests for colorectal cancer screening. J Gastroenterol. 2010 Jul;45(7):703-12

20. Fernández-Bañares F, Alsius A, Campo R, et al. Valor del test inmunoquimico fecal para hemoglobina humana en la priorizacion de la colonoscopia diagnostica [Value of the immunochemical fecal test for human haemoglobin in the prioritization of diagnostic colonoscopy]. Gastroenterol Hepatol 2013; 36:172.

**3. METHODOLOGY**

**Patients**

All patients in the fast-track colonoscopy programs for CRC in the 3 participating hospitals will be included. Health areas of these hospitals comprise a population of about 850,000 inhabitants (Department of Health, 2007). Patients with high-risk symptoms for CRC are send for a fast-track 2-weeks wait full colonoscopy following the government’s program to expedite the diagnosis of CRC. All the diagnostic colonoscopies in the Health Areas involved are performed in the endoscopic units of the three centres. More than 3,000 colonoscopies/year are done in each endoscopy unit. Fast-track colonoscopy may be requested from Primary Care (GPs or gastroenterology specialists) or from the hospital (different medical and surgical specialties). On the other hand, a group of patients with a non-fast track colonoscopy request (performed in less than 2 months) and with a negative FIT will also be included.

Patients with poor preparation for colonoscopy (unless advanced colonic neoplasm –ACN- is diagnosed) will be excluded. Clear instruction sheets will be given to optimize preparation for colonoscopy.

**Interventions**

In all patients the following interventions will be performed:

- FIT in three stool samples prior to colonoscopy. For that we will contact the primary care doctors through a case manager in each hospital (a trained nurse staff hired specifically for the project). This manager will also contact the patient by phone in order to ensure the proper collection of faecal samples in the three different days. In the case of rectal bleeding, patients will be instructed if possible to collect faecal samples on days that they do not have rectal bleeding.

Faecal haemoglobin will be considered positive (qualitative result) for values above 50 mg/L buffer (4 mg Hb/g faeces) (iFOB Linear Leti, Spain) in at least 1 of the three samples. Quantitative values are recorded for each of the three samples from 50 to higher than 2000 mg/L.

- A patient consultation questionnaire including a detailed assessment of colorectal symptoms at the time of the colonoscopy indication, personal and family history of polyps/CRC, smoking and consumption of drugs (NSAIDs, aspirin, anticoagulants). It will be done by means of a structured survey, by phone or in-person, conducted by the case manager of each participating hospital.

- Full colonoscopy with iv sedation as is routinely performed. Colon biopsies and/or polypectomy will be performed if necessary in each patient. The cleaning degree (Boston scale), results of the exploration and pathology studies will be registered. In the case of multiple polyps, the number of polyps, the size of the largest and the most advanced pathology will be registered. ACN will be defined as the presence of CRC or advanced adenoma (AA) (>1 cm or high-grade dysplasia or villous components or 3 or more adenoma). If CRC is recognized, its stage (TNM classification), and location (right, transverse or left colon) will be registered.

**Ethics**

Patients will be informed through a patient information sheet outlining the details of the project. Completion of the patient consultation questionnaire will be entirely on a voluntary basis in compliance with the ethics committees of the participating hospitals. Ethical approval of the study has been obtained.

**Statistical analysis**

The statistical analysis will be developed in three sections: a) Descriptive analysis, b) selection of the best predictive model and c) validation of the model.

a) Descriptive statistics: For quantitative variables the mean, median, standard error and interquartile range will be reported. The 95% confidence intervals will be reported for quantitative variables as well as for proportions.

b) Selection of the best predictive model: Since the result extracted from the questionnaire is based in three possible outcomes: 1) colorectal cancer, 2) advanced adenomas, and 3) no evidence of advanced colonic neoplasm, a multinomial logistic regression (MLR) model will be used to assess the best predictive model for these outcomes taking into account the questionnaire variables (1). This regression model which generalizes logistic regression by allowing more than two discrete outcomes, will be used to predict placement or the probability of outcome for each individual considered taking into account multiple independent variables (1). All models tested will always include sex, age and FIT variables among others. Variable’s selection will be done using a stepwise procedure (2) in the forward direction, adding new variables at each step and removing those which are not statistically significant. The model selection will be done assessing the impact on the likelihood of two subsequent models (2) and looking for the most parsimonious model. The odds ratio (OR) and its 95% confidence interval associated to each variable will be calculated. In this line, the accuracy rate of the predictive score (2) for each individual will be calculated making use of the coefficients of each variable considered.

The presence of advanced colonic neoplasm will be assessed through the comparison between the predictive strategy based on a MLR model and ROC curves (3) based on sensitivity, specificity and positive predictive and negative predictive values.

b.1) Sample size: In the previous study it was observed that the frequency of advanced colonic neoplasm was between 25-30% whereas positive FIT was between 80-90% (4). In this line, using a sample of N=600 patients allows identifying between 150-180 advanced colonic neoplasm patients. Since the minimum number of cases per variable is 10 (1) these figures are adequate to perform the MLR including between 15-18 variables in the final model selected. On the other hand, a sample of 100 patients with non-fast track colonoscopies and negative FIT will be also included as control group. In total, N1=600+100=700 patients will be included in the analysis.

c) Validation of the predictive model: A sample of N2=600 new patients from 3 different centers will be used to assess the predictive performance of the model selected in b). Cohen’s kappa statistic (5) will be used to compare the agreement between the predictive model and the current method in the classification of patients. In addition sensitivity, specificity and positive predictive and negative predictive values will be calculated. A cost-effectiveness study will be also carried out taking into account the cost of a colonoscopy and the number of colonoscopies required to diagnose an advanced colonic neoplasm depending on the predictive strategy.

References

1. Hosmer, D. W., & Lemeshow, S. (1989). Applied logistic regression. New York: Wiley.
2. Efroymson, MA (1960) "Multiple regression analysis." In Ralston, A. and Wilf, HS, editors, Mathematical Methods for Digital Computers. Wiley.
3. Hanley JA, McNeil BJ. The meaning and use of the area under a receiver operating characteristic (ROC) curve. Radiology 1982;143:29-36
4. Fernández-Bañares, F et al. Valor del test inmunoquimico fecal para hemoglobina humana en la priorizacion de la colonoscopia diagnostica [Value of the immunochemical fecal test for human haemoglobin in the prioritization of diagnostic colonoscopy]. Gastroenterol Hepatol 2013; 36:172.
5. Cohen, J (1960). "A coefficient of agreement for nominal scales". Educational and Psychological Measurement 20 (1): 37–46.

**4. DESCRIPTION OF THE PROJECT'S SCIENTIFIC AND SOCIAL INTEREST**

There are long waiting lists for colonoscopy in almost all hospitals of the public health system in Catalunya. From 2005 patients with high-risk clinical symptoms for CRC are send for a fast-track 2-weeks wait full colonoscopy following the government’s program to expedite the diagnosis of CRC. However, the poor specificity of the patient clinical symptoms has resulted in overwhelming referral rates, and the detection rate of cancer is low. Nowadays, this situation has worsened because of present limited resources.

The present research project aims to evaluate if the qualitative and quantitative results of FIT for occult blood in three faecal samples may add specificity to standard evaluation of patient clinical symptoms. The main objective is to derive a predictive score of advanced colonic neoplasm. This score will be a useful tool to prioritize diagnostic colonoscopy and to take decisions in the fast-track programs of colorectal cancer diagnosis.

The findings of the present study will improve the present clinical practice in the early diagnosis of CRC, and also they will promote an adequate use of resources using the best cost-effective strategy to detect advanced colonic neoplasms in symptomatic patients.
