# Supplementary material for: Prediction of advanced colonic neoplasm in symptomatic patients: a scoring system to prioritize colonoscopy (COLONOFIT study)
Source: BMC Cancer. 2019 Jul 25;19:734. doi: 10.1186/s12885-019-5926-4 (PMC6659265; doi:10.1186/s12885-019-5926-4)
Supplement: Supplementary file 2 — FIT performance. (DOCX 12 kb) [file 12885_2019_5926_MOESM2_ESM.docx]

**Supplement 2: i-FOB Linear performance**

Each participant collected three faecal samples on three different days using the Plus-Sed (Linear Chemicals SL, Barcelona, Spain) specimen collection device at room temperature, according to the supplier’s instructions. The device collects 19.9 mg faeces into a 1.6 mL buffer. Samples were analysed using the Kroma iT (Linear Chemicals SL, Barcelona, Spain) analyser. The reagent used for the faecal haemoglobin detection was CLONATEST i-FOB Turbidimetric (Linear Chemicals SL). The detection limit of the test was 1.5 µg Hb/g of faeces and the quantification limit measured in our laboratory was 4 µg Hb/g of faeces. The analyser was calibrated once a week with the calibrators provided by the manufacturer. The adjustment of the calibration curve was made according a logit/log4 model. The analysis of the quality control materials (i-FOB NI and NII at concentrations of 12 µg/g and 40 µg/g) was performed every 100 samples and accepted in the range of ±2 standard deviations. The coefficients of variation obtained were 6% and 8%, respectively. The method calibration curve was linear for concentrations in the range 4-80 µg/g. The samples over this range were automatically diluted 1/3 and if they still were over the range, they were informed as >160 µg/g. A control reading was done 20 seconds after the reaction had started to detect a Hook effect or prozone. If a quick reaction was detected, the sample was automatically diluted up to 1/10.

The results were recorded electronically and then automatically sent to the laboratory information system.
