# Supplementary material for: Prediction of advanced colonic neoplasm in symptomatic patients: a scoring system to prioritize colonoscopy (COLONOFIT study)
Source: BMC Cancer. 2019 Jul 25;19:734. doi: 10.1186/s12885-019-5926-4 (PMC6659265; doi:10.1186/s12885-019-5926-4)
Supplement: Supplementary file 3 — Supplementary statistical methods. (DOCX 12 kb) [file 12885_2019_5926_MOESM3_ESM.docx]

**Supplementary statistical methods**

**Validation cohort: sample size calculation**

Once we had a better estimate of the prevalence of ACN in our population and the best set of predictive variables for the ACN, several random samples of variable size (from 400 to 1000 individuals) were extracted from the development database and calculated the diagnostic validity indices for each sample size. It was observed that from sizes of 500 individuals the C-Statistic was stabilized around the values obtained in the development phase. Thus, we settled that a minimum sample size of 500 was required for the validation phase.

**Derivation of the FIT variables included in the model: Selection of a cut-off for MAXFIT variable.**

MAXFIT was introduced as a categorical variable. In order to find out what thresholds were able to induce a partition that separate the individuals in relation to their risk of ACN (low, middle, high) we tried two strategies. First of all, following a ROC analysis, we plotted Youden index against f-Hb values and we found the threshold was placed at the boundary of the parameter range, which is not appropriate to distinguish between a number of risk levels. Secondly, the diagnostic odds ratio (DOR) plot against the values of f-Hb showed two maximums that indicated which cut-offs separated the different levels of risk (see Figure). The two maximums were located at 11 and 32 µg Hb/g faeces, but we selected as cut-off 11 µg Hb/g faeces since that was the minimum MAXFIT among all CRCs in the study phase 1.

Figure. Values of the expected Diagnostic Odds Ratio as a function of the MAXFIT variable in comparison with Youden index.
