# Supplementary material for: Prediction of advanced colonic neoplasm in symptomatic patients: a scoring system to prioritize colonoscopy (COLONOFIT study)
Source: BMC Cancer. 2019 Jul 25;19:734. doi: 10.1186/s12885-019-5926-4 (PMC6659265; doi:10.1186/s12885-019-5926-4)
Supplement: Supplementary file 5 — Figure S1. Tumour localization and staging in both study phases. (DOCX 11 kb) [file 12885_2019_5926_MOESM5_ESM.docx]

Figure S1. Tumour localization and staging in both study phases.
