# Supplementary material for: Prediction of advanced colonic neoplasm in symptomatic patients: a scoring system to prioritize colonoscopy (COLONOFIT study)
Source: BMC Cancer. 2019 Jul 25;19:734. doi: 10.1186/s12885-019-5926-4 (PMC6659265; doi:10.1186/s12885-019-5926-4)
Supplement: Supplementary file 6 — Table S1 Indication for colonoscopy in both study phases. (DOCX 12 kb) [file 12885_2019_5926_MOESM6_ESM.docx]

**SUPPLEMENTARY FILE 6**

**Table suppl 1.** Indication for colonoscopy in both study phases.

| **NICE criteria** | **Phase 1**  **(n=867)** | **Phase 2**  **(n=628)** |
| --- | --- | --- |
| Age 40 and over with unexplained weight loss and abdominal pain | 155 (17.8%) | 80 (12.7%) |
| Age 50 and over with unexplained rectal bleeding | 148 (17%) | 131 (20.8%) |
| Age 60 and over with iron-deficiency anaemia | 156 (18%) | 161 (25.6%) |
| Age 60 and over with changes in their bowel habit | 301 (34.7%) | 265 (42.1%) |
| Age under 50 with rectal bleeding and abdominal pain | 56 (6.4%) | 12 (1.9%) |
| Age under 50 with rectal bleeding and change in bowel habit | 60 (6.9%) | 19 (3%) |
| Age under 50 with rectal bleeding and weight loss | 24 (2.7%) | 8 (1.3%) |
| Age under 50 with rectal bleeding and iron-deficiency anaemia | 14 (1.6%) | 7 (1.1%) |
| Rectal or abdominal mass or enema/CT/MR suspicion | 67 (7.7%) | 38 (6%) |
| Unexplained GI symptoms with positive FIT | 96 (11.1%) | 46 (7.3%) |
| No NICE criteria for a fast-track colonoscopy | 136 (15.7%) | 101 (16.1%) |
