# Supplementary material for: Prediction of advanced colonic neoplasm in symptomatic patients: a scoring system to prioritize colonoscopy (COLONOFIT study)
Source: BMC Cancer. 2019 Jul 25;19:734. doi: 10.1186/s12885-019-5926-4 (PMC6659265; doi:10.1186/s12885-019-5926-4)
Supplement: Supplementary file 7 — Table S2 Description of patients included in study phase 1 and 2. (DOCX 22 kb) [file 12885_2019_5926_MOESM7_ESM.docx]

**Supplementary file 7.**

**Table suppl 2:** Description of patients included in study phase 1 and 2.

|  | **Derivation Cohort (N=867)** | | |  | **Validation Cohort (N=628)** | | |  |
| --- | --- | --- | --- | --- | --- | --- | --- | --- |
|  |  | | |  |  | | |  |
| X | **Control** | **AA** | **CCR** |  | **Control** | **AA** | **CCR** | **p-value*** |
|  | N=696 | N=104 | N=67 |  | N=480 | N=99 | N=49 |  |
| Gender: |  |  |  |  |  |  |  | 0.092 |
| Man | 311 (44.7%) | 58 (55.8%) | 45 (67.2%) |  | 224 (46.8%) | 59 (59.6%) | 30 (61.2%) |  |
| Woman | 385 (55.3%) | 46 (44.2%) | 22 (32.8%) |  | 256 (53.2%) | 40 (40.4%) | 19 (38.8%) |  |
| Age group (years): |  |  |  |  |  |  |  | 0.421 |
| ≤40 | 66 (9.48%) | 1 (0.96%) | 0 (0.00%) |  | 15 (3.33%) | 0 (0.00%) | 1 (2.04%) |  |
| 40-50 | 98 (14.1%) | 6 (5.77%) | 0 (0.00%) |  | 57 (11.9%) | 2 (2.02%) | 1 (2.04%) |  |
| 50-60 | 157 (22.6%) | 25 (24.0%) | 9 (13.4%) |  | 86 (17.9%) | 19 (19.2%) | 3 (6.12%) |  |
| >60 | 375 (53.9%) | 72 (69.2%) | 58 (86.6%) |  | 322 (66.9%) | 78 (78.8%) | 44 (89.8%) |  |
| Body mass index (Kg/m^2^): |  |  |  |  |  |  |  | 0.081 |
| ≤25 | 246 (35.3%) | 30 (28.8%) | 17 (25.4%) |  | 156 (32.6%) | 29 (29.3%) | 17 (34.7%) |  |
| >25 | 450 (64.7%) | 74 (71.2%) | 50 (74.6%) |  | 324 (67.4%) | 70 (70.7%) | 32 (65.3%) |  |
| MAXFIT (out of 3 samples): |  |  |  |  |  |  |  | 0.009 |
| ≤4 | 441 (63.4%) | 24 (23.1%) | 0 (0.00%) |  | 336 (70.0%) | 35 (35.4%) | 3 (6.12%) |  |
| (>4-11) | 67 (9.63%) | 9 (8.65%) | 0 (0.00%) |  | 27 (5.62%) | 5 (5.05%) | 2 (4.08%) |  |
| >11 | 188 (27.0%) | 71 (68.3%) | 67 (100%) |  | 117 (24.4%) | 59 (59.6%) | 44 (89.8%) |  |
| Colonoscopy (5 years before): |  |  |  |  |  |  |  | 0.079 |
| No | 555 (79.7%) | 90 (86.5%) | 63 (94.0%) |  | 381 (79.4%) | 88 (88.9%) | 47 (95.9%) |  |
| Yes | 141 (20.3%) | 14 (13.5%) | 4 (5.97%) |  | 99 (20.6%) | 11 (11.1%) | 2 (4.08%) |  |
| Smoking (History): |  |  |  |  |  |  |  | 0.081 |
| No | 359 (51.6%) | 46 (44.2%) | 31 (46.3%) |  | 232 (48.4%) | 43 (43.4%) | 26 (53.1%) |  |
| Yes (Ex-smoker or current) | 337 (48.4%) | 58 (55.8%) | 36 (53.7%) |  | 248 (51.6%) | 56 (56.6%) | 23 (46.9%) |  |
| Smoking (Years): |  |  |  |  |  |  |  | 0.189 |
| No | 359 (51.6%) | 46 (44.2%) | 31 (46.3%) |  | 232 (48.4%) | 43 (43.4%) | 26 (53.1%) |  |
| ≤42 | 191 (27.4%) | 19 (18.3%) | 6 (8.96%) |  | 118 (24.5%) | 14 (14.1%) | 2 (4.08%) |  |
| >42 | 146 (21.0%) | 39 (37.5%) | 30 (44.8%) |  | 130 (27.0%) | 42 (42.4%) | 21 (42.9%) |  |
| Drugs (NSAIDs/ antiplatelet agents/anticoagulants): |  |  |  |  |  |  |  | 0.077 |
| No | 415 (59.6%) | 51 (49.0%) | 47 (70.1%) |  | 295 (61.5%) | 57 (57.6%) | 39 (79.6%) |  |
| Yes | 281 (40.4%) | 53 (51.0%) | 20 (29.9%) |  | 185 (38.5%) | 42 (42.4%) | 10 (20.4%) |  |
| Anticoagulants: |  |  |  |  |  |  |  | 0.121 |
| No | 673 (96.7%) | 94 (90.4%) | 62 (92.5%) |  | 442 (92.1%) | 86 (86.9%) | 47 (95.9%) |  |
| Yes | 23 (3.30%) | 10 (9.62%) | 5 (7.46%) |  | 38 (7.90%) | 13 (13.1%) | 2 (4.08%) |  |
| NSAIDs: |  |  |  |  |  |  |  | 0.092 |
| No | 525 (75.4%) | 73 (70.2%) | 63 (94.0%) |  | 389 (81.1%) | 78 (78.8%) | 46 (93.9%) |  |
| Yes | 171 (24.6%) | 31 (29.8%) | 4 (5.97%) |  | 91 (18.9%) | 21 (21.2%) | 3 (6.12%) |  |
| Abdominal pain: |  |  |  |  |  |  |  | 0.104 |
| No | 299 (43.0%) | 57 (54.8%) | 40 (59.7%) |  | 245 (51.1%) | 51 (51.5%) | 27 (55.1%) |  |
| Yes | 397 (57.0%) | 47 (45.2%) | 27 (40.3%) |  | 235 (48.9%) | 48 (48.5%) | 22 (44.9%) |  |
| Iron deficiency: |  |  |  |  |  |  |  | 0.1157 |
| No | 237 (34.1%) | 44 (42.3%) | 12 (17.9%) |  | 329 (68.6%) | 59 (59.6%) | 23 (46.9%) |  |
| Yes | 174 (25.0%) | 21 (20.2%) | 34 (50.7%) |  | 104 (21.6%) | 33 (33.3%) | 24 (49.0%) |  |
| Unknown | 285 (40.9%) | 39 (37.5%) | 21 (31.3%) |  | 47 (9.77%) | 7 (7.07%) | 2 (4.08%) |  |
| Iron deficiency anaemia: |  |  |  |  |  |  |  | 0.075 |
| No | 371 (53.3%) | 57 (54.8%) | 20 (29.9%) |  | 347 (72.3%) | 63 (63.6%) | 17 (34.7%) |  |
| Yes | 166 (23.9%) | 20 (19.2%) | 34 (50.7%) |  | 128 (26.6%) | 35 (35.4%) | 32 (65.3%) |  |
| Unknown | 159 (22.8%) | 27 (26.0%) | 13 (19.4%) |  | 5 (1.04%) | 1 (1.01%) | 0 (0.00%) |  |
| Type change bowel habits: |  |  |  |  |  |  |  | 0.106 |
| No | 261 (37.5%) | 49 (47.1%) | 29 (43.3%) |  | 196 (41.0%) | 42 (42.4%) | 21 (42.9%) |  |
| Diarrhoea | 226 (32.5%) | 28 (26.9%) | 31 (46.3%) |  | 176 (36.6%) | 35 (35.4%) | 19 (38.8%) |  |
| Constipation | 179 (25.7%) | 24 (23.1%) | 7 (10.4%) |  | 97 (20.2%) | 19 (19.2%) | 8 (16.3%) |  |
| Unknown | 30 (4.31%) | 3 (2.88%) | 0 (0.00%) |  | 11 (2.29%) | 3 (3.03%) | 1 (2.04%) |  |
| Abdominal pain duration: |  |  |  |  |  |  |  | 0.098 |
| No | 299 (43.0%) | 57 (54.8%) | 40 (59.7%) |  | 245 (51.1%) | 51 (51.5%) | 27 (55.1%) |  |
| Yes 1-12 days | 123 (17.7%) | 13 (12.5%) | 13 (19.4%) |  | 63 (13.1%) | 17 (17.2%) | 6 (12.2%) |  |
| Yes >12 days | 274 (39.4%) | 34 (32.7%) | 14 (20.9%) |  | 172 (35.8%) | 31 (31.3%) | 16 (32.7%) |  |
| Rectal bleeding pattern: |  |  |  |  |  |  |  | 0.069 |
| No | 342 (49.1%) | 52 (50.0%) | 32 (47.8%) |  | 290 (60.5%) | 59 (59.6%) | 27 (55.1%) |  |
| Yes, without specific pattern | 219 (31.5%) | 28 (26.9%) | 15 (22.4%) |  | 117 (24.3%) | 27 (27.3%) | 9 (18.4%) |  |
| Yes, with specific pattern | 135 (19.4%) | 24 (23.1%) | 20 (29.9%) |  | 73 (15.2%) | 13 (13.1%) | 13 (26.5%) |  |
| Rectal bleeding days: |  |  |  |  |  |  |  | 0.062 |
| No Rectal bleeding | 342 (49.1%) | 52 (50.0%) | 32 (47.8%) |  | 290 (60.5%) | 59 (59.6%) | 27 (55.1%) |  |
| ≤30 | 328 (47.1%) | 48 (46.2%) | 26 (38.8%) |  | 179 (37.2%) | 39 (39.4%) | 18 (36.7%) |  |
| >30 | 26 (3.74%) | 4 (3.85%) | 9 (13.4%) |  | 11 (2.29%) | 1 (1.01%) | 4 (8.16%) |  |
| NSAMPLES>4 (Number of samples with FIT>4): |  |  |  |  |  |  |  | 0.003 |
| 0 | 441 (63.4%) | 24 (23.1%) | 0 (0.00%) |  | 336 (70.0%) | 35 (35.4%) | 3 (6.12%) |  |
| 1 | 110 (15.8%) | 14 (13.5%) | 4 (5.97%) |  | 53 (11.0%) | 13 (13.1%) | 2 (4.08%) |  |
| 2 | 71 (10.2%) | 14 (13.5%) | 4 (5.97%) |  | 45 (9.38%) | 13 (13.1%) | 9 (18.4%) |  |
| 3 | 74 (10.6%) | 52 (50.0%) | 59 (88.1%) |  | 46 (9.58%) | 38 (38.4%) | 35 (71.4%) |  |
| Haemoglobin (g/dL): |  |  |  |  |  |  |  | 0.126 |
| ≤11 | 105 (15.1%) | 17 (16.3%) | 26 (38.8%) |  | 67 (14.1%) | 8 (8.08%) | 14 (28.6%) |  |
| >11 | 481 (69.1%) | 77 (74.0%) | 35 (52.2%) |  | 395 (82.1%) | 87 (87.9%) | 34 (69.4%) |  |
| Unknown | 110 (15.8%) | 10 (9.62%) | 6 (8.96%) |  | 18 (3.74%) | 4 (4.04%) | 1 (2.04%) |  |
| Ferritin (ng/mL): |  |  |  |  |  |  |  | 0.113 |
| ≤40 | 176 (25.3%) | 23 (22.1%) | 33 (49.3%) |  | 128 (26.8%) | 31 (31.3%) | 23 (46.9%) |  |
| >40 | 225 (32.3%) | 40 (38.5%) | 13 (19.4%) |  | 227 (47.2%) | 42 (42.4%) | 20 (40.8%) |  |
| Unknown | 295 (42.4%) | 41 (39.4%) | 21 (31.3%) |  | 125 (26.0%) | 26 (26.3%) | 6 (12.2%) |  |
| CEA (ng/mL): |  |  |  |  |  |  |  | 0.109 |
| ≤12 | 610 (87.6%) | 88 (84.6%) | 47 (70.1%) |  | 392 (81.7%) | 82 (82.8%) | 37 (75.5%) |  |
| >12 | 5 (0.72%) | 1 (0.96%) | 10 (14.9%) |  | 5 (1.04%) | 1 (1.01%) | 11 (22.4%) |  |
| Unknown | 81 (11.6%) | 15 (14.4%) | 10 (14.9%) |  | 83 (17.3%) | 16 (16.2%) | 1 (2.04%) |  |

***p-value**: differences between Derivation (Phase I) and Validation (Phase II) cohorts (obtained through a logistic regression model). **MAXFIT**: maximum f-Hb value; **NSAMPLES>4**: Number of samples with FIT >4 μg Hb/g faeces.
